# 

**Published:** 1857-03

**Authors:** 


### CONTENTS.

| | Page. |
|---|---|
| **Original Communications—** | |
| A Successful Case of Ovariotomy. By Samuel Long, M.D. | 103 |
| Valvular Aortic Constrictive Disease. By F. B. Norcom, M.D. | 108 |
| Chlorate of Potassa in Typhoid Fever. By E. C. Ellet, M.D. | 112 |
| Veratrum Viride in Fevers. By A. W. Dewey, M.D. | 115 |
| **Selections—** | |
| On the General Use of Chloroform. By Dr. M'Leod | 119 |
| Is Strychnia Cumulative in the System?—Does it Affect the Encephalon? By W. G. Thomas, M.D. | 125 |
| Dr. Edward Brown-Sequard's Experimental and Clinical Researches applied to Physiology and Pathology | 127 |
| **Book Notices** | 133 |
| **Medical Intelligence** | 139 |
| **Editorial** | 143 |

